# Proinflammatory Microenvironment During *Kingella kingae* Infection Modulates Osteoclastogenesis

**DOI:** 10.3389/fimmu.2021.757827

**Published:** 2021-12-02

**Authors:** Ayelén Ivana Pesce Viglietti, Franco Agustín Sviercz, Cinthya Alicia Marcela López, Rosa Nicole Freiberger, Jorge Quarleri, María Victoria Delpino

**Affiliations:** Instituto de Investigaciones Biomédicas en Retrovirus y Sida (INBIRS), Universidad de Buenos Aires, Consejo Nacional de Investigaciones Científicas y Técnicas (CONICET), Buenos Aires, Argentina

**Keywords:** inflammation, osteoclastogenesis and bone loss, *Kingella kingae*, TNF-α, IL-1β

## Abstract

*Kingella kingae* is an emerging pathogen that causes septic arthritis, osteomyelitis, and bacteremia in children from 6 to 48 months of age. The presence of bacteria within or near the bone is associated with an inflammatory process that results in osteolysis, but the underlying pathogenic mechanisms involved are largely unknown. To determine the link between *K. kingae* and bone loss, we have assessed whether infection *per se* or through the genesis of a pro-inflammatory microenvironment can promote osteoclastogenesis. For that purpose, we examined both the direct effect of *K. kingae* and the immune-mediated mechanism involved in *K. kingae*-infected macrophage-induced osteoclastogenesis. Our results indicate that osteoclastogenesis is stimulated by *K. kingae* infection directly and indirectly by fueling a potent pro-inflammatory response that drives macrophages to undergo functional osteoclasts *via* TNF-α and IL-1β induction. Such osteoclastogenic capability of *K. kingae* is counteracted by their outer membrane vesicles (OMV) in a concentration-dependent manner. In conclusion, this model allowed elucidating the interplay between the *K. kingae* and their OMV to modulate osteoclastogenesis from exposed macrophages, thus contributing to the modulation in joint and bone damage.

## Introduction

*Kingella kingae* is a common etiology agent of septic arthritis, osteomyelitis, and bacteremia in children from 6 to 48 months of age. This bacteria is an emerging pediatric pathogen characterized by asymptomatic pharyngeal colonization that could be followed by bacterial translocation across the pharyngeal epithelial barrier with dissemination to distant locations, including infective endocarditis, bacteremia, septic arthritis, and osteomyelitis (1, 2). With the notorious exception of endocardial invasion, many patients are afebrile or have a mildly elevated body temperature and are in good general condition, and the yield of blood cultures is low, suggesting that the bacteremic stage of the disease is transient (1–3).

The bacterial infection localized in bones or contiguous tissue is a serious complication that causes bone loss. The presence of bacteria within or near the bone is associated with an inflammatory process that results in osteolysis, and *K. kingae* appears not to be an exception (4–6). The number of children colonized by this bacteria is more than those with clinical disease (7). Its infection can produce occult bacteremia, lower respiratory tract manifestations, and ocular infections. However, the major manifestation of *K. kingae* disease is represented by skeletal system infections (8). Patients treated with antibiotics without surgical intervention have frequently favorable outcomes, and even for those who were not treated at all, the need for extended intravenous treatment or surgical intervention seems very low (9). Self-limited involvement of the joints and/or bones in the course of a *K. kingae* bacteremic episode has also been reported, suggesting an abortive clinical course (2, 9–12).

A different virulence factor has been associated to *K. kingae* disease (8). Among these, *K. kingae* secretes the RtxA toxin that belongs to the family of Repeats in ToXin (RTX) cytotoxins that are produced by bacteria (13, 14). This is the main virulence factor identified, and its role as a virulence factor has been demonstrated in animal models of infection (13, 15).

*K. kingae* is protected from host immune response due to the presence of a capsule, which facilitates mucosal colonization and survival during bloodstream invasion (16–18). The attachment of the bacteria to the respiratory tract is possible due to the presence of type IV pili and KnnH adhesion (16). Bacteria use specialized secretion systems to deliver virulence factors (19). However, in addition to these systems, some bacteria release outer membrane vesicles (OMV) that act as a mechanism for the delivery of virulence factors to the host. In previous studies, it has been demonstrated that *K. kingae* produced OMV that could play a role in bacterial pathogenesis (4). The role of this OMV in bone pathology started to be elucidated. These studies revealed that OMV could be internalized by osteoblasts and synoviocytes, inducing the secretion of inflammatory mediators (4).

It is now recognized that the formation of osteoclasts is centered on the key osteoclastogenic cytokine, the receptor activator of NF-*κ*B ligand (RANKL). The balance between RANKL and osteoprotegerin (its decoy receptor) in osteoblast determines the differentiation of new osteoclast under physiological conditions (20), but the cellular recruitment in chronic inflammatory bone disease, in particular B and T lymphocytes, neutrophils, dendritic cells, and macrophages, contributes to bone resorption. In this setting, not only RANKL but also proinflammatory cytokines, such as TNF-α, IL-1β, and IL-6, are important in the development of the disease and bone loss (20–25). Bone resorption is accelerated by RANKL and cytokines produced by T and B lymphocytes (26–28). During inflammation, macrophages can contribute to bone resorption through the production of proinflammatory cytokines and also by differentiating into osteoclasts (28, 29).

To determine a link between *K. kingae* infection and loss of bone, we tested the hypothesis that the infection might create a microenvironment that would promote the generation of osteoclasts, the only cells known so far to be able to degrade bone. For that purpose, we examined the immune-mediated mechanisms of osteoclastogenesis triggered by *K. kingae*-infected macrophages. Moreover, as *K. kingae* effects on eukaryotic cells can be caused by a combination of virulence factors, we aim to investigate the direct effect and global impact of *K. kingae* on osteoclastogenesis.

This model allowed us to elucidate, at the single-cell-type level, the ability of *K. kingae* to modulate the osteoimmune response contributing to the observed bone damage and also to bacterial persistence.

## Materials and Methods

### Bacterial Culture

*K. kingae* strain was isolated from the joint fluid of a patient attended to at the Hospital de Clínicas “José de San Martín”, Buenos Aires, Argentina. It was grown on chocolate agar (Chocolate II Agar; BD, Franklin Lakes, NJ) or Brain Heart Infusion broth (BHI) with 5% of hemoglobin at 37°C with 5% CO_2_. The stocks were stored at -80°C in BHI with 30% of glycerol. The bacterial number in culture was estimated by comparing optical densities at 600 nm with a standard curve obtained in our laboratory.

To obtain the standard curve, the spectrophotometer was calibrated using BHI as the blank reference. A single colony of *K. kingae* was inoculated into 1 ml of BHI and then incubated at 37°C with 5% CO_2_ for 48 h. Then, serial dilutions of the inoculum were performed and used to determine the OD600, and another aliquot was plated on chocolate agar plates and incubated overnight at 37°C with 5% CO_2_ to determine the colony-forming unit counts.

To prepare the inoculum, cultures were diluted in sterile phosphate-buffered saline (PBS) to the desired bacterial concentration.

To obtain heat-killed *K. kingae* (HKKk), the bacteria were washed five times for 10 min each in sterile PBS, heat-killed at 60°C for 1 h, aliquoted, and stored at -70°C until they were used. The total absence of *K. kingae* viability after heat killing was verified by the absence of bacterial growth on chocolate agar.

All experiments were performed in BSL-2 and BSL-3 facilities at the INBIRS.

### Isolation of Outer Membrane Vesicles

*K. kingae* was grown on chocolate agar plates for 72 h, and the bacteria were scraped from plates and harvested by centrifugation at 6,000 × *g*. The cell-free supernatant was sterilized through a 0.22-µm-pore-size filter. Then, the supernatant was ultracentrifuged at 150,000 × *g* for 6 h at 4°C to pellet the vesicles. The supernatant was removed, and the pellets were resuspended in PBS with 20% glycerol and 2 mM CaCl_2_. The protein concentration was measured using a bicinchoninic acid assay (Pierce). The OMV were stored at -80°C. Between 9 to 14 µg of OMV was obtained from 1 × 10^10^ bacteria.

### Cellular Infection and HKKk Stimulation

Unless otherwise specified, all experiments were performed at 37°C in 5% CO_2_ atmosphere. THP-1 cells (human monocytic cell line) were obtained from the American Type Culture Collection (Manassas, VA) and grown in RPMI 1640 supplemented with 10% heat-inactivated fetal bovine serum (Gibco-BRL, Life Technologies, Grand Island, NY), 100 U/ml of penicillin, and 100 mg/ml of streptomycin (complete medium). To induce maturation, the cells were cultured in the presence of 150 U/ml of 1,25-dihydroxyvitamin D3 (Endogen) for 72 h. For the experiments, 5 × 10^5^ cells/ml were stimulated with HKKk at a final concentration of 1 × 10^5^ and 1 × 10^6^ bacteria/ml or infected with *K. kingae* at multiplicities of infection of 1 and 10 for 2 h in medium without antibiotics. Then, the cells were extensively washed to remove extracellular bacteria, and the infected cells were maintained for an additional 24 h in a complete medium with 50 μg/ml of gentamicin. The supernatants were harvested and stored at -80°C for the determination of cytokine and osteoclastogenesis experiments. The viability of THP-1 cells after the addition of *K. kingae* (measured using trypan blue exclusion test) was preserved between 90 to 95% irrespective of the inoculum used.

### Measurement of Cytokine Concentrations in Culture Supernatants

The secretion of human and mouse IL-1β, IL-6, and TNF-α in the supernatants was quantified with an ELISA kit (BD PharMingen, San Diego, CA, USA) and the production of human and mouse RANKL by an ELISA kit (R&D Systems Minneapolis, MN, USA).

### Osteoclast Formation Assay

RAW 264.7 cells (mouse macrophage), at a concentration of 5 × 10^4^ cells/well, were cultured onto glass coverslips in 24-well plates for 7 days and cultured in a complete medium containing 30 ng/ml macrophage colony-stimulating factor (M-CSF) and 0.2 ml of culture supernatants from *K. kingae*-infected THP-1 cells. As positive controls of osteoclast formation, the RAW 264.7 cell cultures received 50 ng/ml murine RANKL. On day 3, the culture medium and all reagents were replaced. To identify osteoclasts, the cells were fixed in 4% paraformaldehyde and stained for tartrate-resistant acid phosphatase (TRAP) (Sigma-Aldrich, St. Louis, MO, USA). TRAP-positive, multinucleated (more than three nuclei) cells were defined as osteoclasts, and the number was determined by microscopic counts. For each well, TRAP-positive multinucleated cells were calculated by counting their number in five microscopic fields (×20).

### Direct Effect of *K. kingae* and Its OMV on Osteoclastogenesis

RAW264.7 cells were infected with *K. kingae* at multiplicity of infection (MOI) = 1 or stimulated with HKKk (1 × 10^5^ bacteria/ml) in a complete medium without antibiotics containing 30 ng/ml M-CSF. Alternatively, RAW264.7 cells were exposed to the bacteria or HKKk during 2 h, then extensively washed with culture medium to remove extracellular bacteria, and incubated in complete medium containing 30 ng/ml M-CSF with 50 μg/ml of gentamicin. To determine the role of OMV, osteoclast differentiation was performed in the presence of HKKk (1 × 10^5^ bacteria/ml) and different amounts of OMV (10, 1, and 0.1 μg/ml.). The presence of TRAP-positive multinucleated cells was determined as was previously described.

### Pit Formation Assay

Osteoclast differentiation assay was performed on hydroxyapatite disks (BD BioCoat Osteologic (BD Biosciences, San Diego, CA, USA) for 9 days. The media and all reagents were replaced every day to avoid the acidification of the medium. After culturing with cells, the hydroxyapatite discs were washed with 1 M NH_4_OH to remove adherent cells. After rinsing with water, the hydroxyapatite discs were visually examined by light microscopy, and the area of resorption was calculated using the NIH Image J analysis software (http://rsbweb.nih.gov/ij/). The results were expressed as the percentage of the total area of the well that appears resorbed.

### Neutralization Experiments

Neutralization experiments were performed using anti-TNF-α-neutralizing antibody (clone MAb1) or its isotype control (both from BD Biosciences, San Diego, CA) and anti-IL-1β-neutralizing antibody (clone AS10) or its isotype control (BD Biosciences). In the neutralization experiments with anti-TNF-α antibody or anti-IL-1β antibody, the conditioned medium was preincubated with the corresponding antibody (or isotype control) for 1 h at 37°C before use.

### MTT Colorimetric Assay

Cell proliferation/viability was measured by MTT colorimetric assay (Sigma-Aldrich, Argentina) at 7 days post-infection or stimulation with HKKk and OMV, according to the instructions of the manufacturer. The absorbance was measured by using a microplate reader at 570 nm. The OD of each well was quantified as a percentage compared with the untreated cells. All experiments were carried out in triplicate.

### Statistical Analysis

Statistical analysis was performed with one-way ANOVA. Multiple comparisons between all pairs of groups were made with Tukey’s post-test, and those against two groups were done with Student᾽s *t*-test and Mann–Whitney test. To determine normality, the Shapiro–Wilk normality test was used. Graphical and statistical analyses were performed with GraphPad Prism 7.0 software. Each experiment was performed in triplicate with different culture preparations on three independent occasions. Data were represented as mean ± SD measured in triplicate from three individual experiments.

## Results

### *K. kingae* Infection Induces Proinflammatory Cytokine Secretion

**Figure 1:**
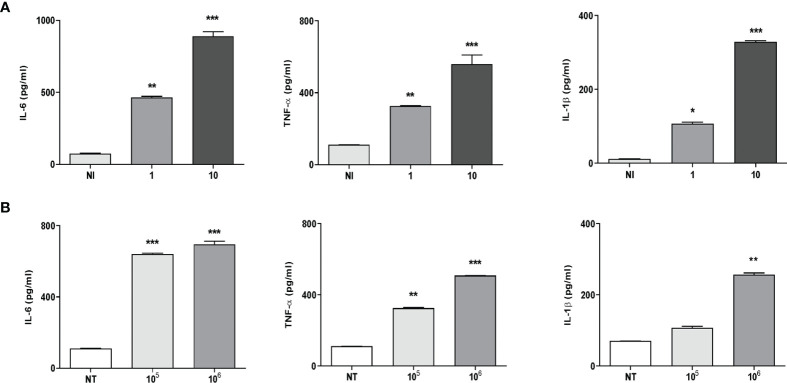
Monocytes produce proinflammatory cytokines in response to *Kingella kingae* infection. THP-1 cells were infected with *K. kingae* at different multiplicities of infection 1 and 10 **(A)** or stimulated with HKKk (1 × 10^5^ and 1 × 10^6^ bacteria/ml **(B)**. IL-6, TNF-α, and IL-1β were determined in culture supernatants by ELISA at 24 h post-infection. NI, non-infected; NT, non-treated. The graphics are showing values obtained from three independent experiments. Data are given as mean ± SD. *P < 0.05; **P < 0.01; ***P < 0.001 *versus* control (NI and NT).

It is known that some proinflammatory cytokines could have the ability to promote or inhibit osteoclast differentiation directly or *via* the RANKL/OPG system (30). Thus, the production of IL-6, TNF-α, IL-1β, and RANKL in culture supernatants from THP-1 monocytes in response to *K. kingae* infection was examined. Infection of human monocytes with *K. kingae* performed at MOIs of 1 and 10 elicited the secretion of IL-6, TNF-α, and IL-1β at 24 h post-infection in an inoculum-dependent manner (**Figure 1A**). On the contrary, RANKL secretion was not detected in monocytes infected with *K. kingae*.

To elucidate whether viable bacteria were necessary to induce proinflammatory cytokine secretion, monocytes were stimulated with HKKk. The production of IL-6, TNF-α, and IL-1β was also markedly increased in culture supernatants from monocytes that were stimulated with HKKk when compared with unstimulated cells (**Figure 1B**). Additionally, the levels of cytokines induced by HKKk were comparable to those produced by similar amounts of live bacteria (**Figure 1**). These results indicate that human monocytes exposed to *K. kingae* can produce proinflammatory cytokines but do not produce RANKL. The ability of HKKk to produce proinflammatory cytokines also suggests that the inflammatory response could be induced by a structural component of the bacteria.

### Culture Supernatants From *K. kingae*-Infected Monocytes Induce Osteoclast Differentiation Mediated by TNF-α and IL-1β

Osteoclasts originate from the fusion of precursors from monocyte/macrophage lineage in the bone marrow (31, 32) and play a key role in bone resorption. The differentiation process could involve soluble mediators belonging to inflammatory cells, such as monocytes/macrophages, in combination with M-CSF (33, 34). The role of IL-6 in the induction of osteoclast differentiation appears to be controversial and mainly indirectly mediated through osteoblast stimulation (30, 35–37). On the contrary, TNF-α and IL-1β have been implicated in RANKL-independent osteoclast formation (30, 34). To determine if soluble mediators secreted by *K. kingae*-infected human monocytes could stimulate osteoclast differentiation from RAW 264.7, these cells were stimulated with M-CSF in the presence of culture supernatants from *K. kingae*-infected human monocytes, and osteoclastogenesis was evaluated by the generation of TRAP-positive multinucleated cells. RANKL was used as a positive control. The production of osteoclast-like cells was generated by culture supernatants from *K. kingae*-infected human monocytes but not by those from uninfected cells.

**Figure 2:**
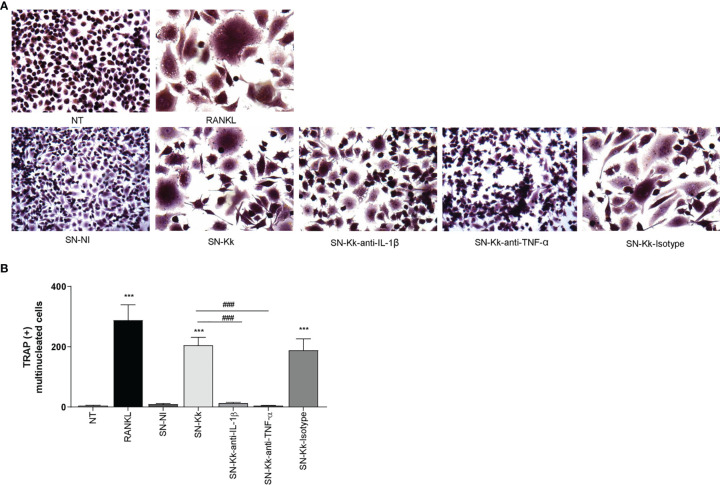
Culture supernatants from *Kingella kingae*-infected monocytes induce osteoclast differentiation mediated by TNF-α and IL-1β. Supernatants from *K. kingae*-infected THP-1 monocytes (multiplicity of infection = 1) induce RAW 264.7-derived osteoclastogenesis. RAW 264.7 cells were either untreated or stimulated with culture supernatants from *K. kingae*-infected THP-1 monocytes (SN-Kk) or with culture supernatants from non-infected THP-1 monocytes (SN-NI) added at a 1:2 proportion in conjunction with macrophage colony-stimulating factor. To inhibit TNF-α and IL-1β, experiments were conducted in the presence of neutralizing antibodies. After 7 days, osteoclastogenesis was determined by the generation of TRAP-positive multinucleated cells (more than three nuclei). Representative digital images were taken by light microscopy **(A)**, and TRAP-positive multinucleated cells were identified and counted **(B)**. RANKL was used as a positive control. NT, non-treated. The graphics are showing values obtained from three independent experiments. Data are given as mean ± SD. ***P < 0.001 *versus* cells treated with SN-NI or NT. ^###^ *P* < 0.001.

To determine the role of TNF-α and IL-1β in the osteoclastogenesis elicited by *K. kingae*, stimulation experiments were performed as described above but in the presence of an anti-TNF-α- or an anti-IL-1β-neutralizing antibody. The TNF-α blocking antibody—but not the isotype control—completely inhibits osteoclastogenesis induced by culture supernatants from monocytes infected by *K. kingae* (**Figure 2**). Osteoclastogenesis was likewise significantly reduced in experiments performed with culture supernatants treated with IL-1β-blocking antibody in which the presence of binucleated cells could also be detected. These results indicate that monocyte-secreted TNF-α and IL-1β induced by *K. kingae* could be involved in the bone resorption observed in patients.

### TNF-α and IL-1β Secreted by *K. kingae*-Infected Monocytes Determine Proinflammatory Cytokine Secretion by Osteoclast

**Figure 3:**
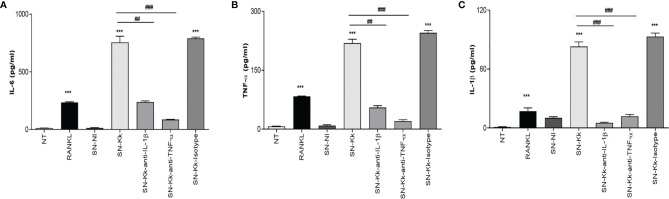
TNF-α and IL-1β secreted by *Kingella kingae*-infected monocytes determine proinflammatory cytokine secretion by osteoclast precursors. Culture supernatants from *K. kingae*-infected THP-1 monocytes at multiplicity of infection = 1 (SN-Kk), pre-incubated or not with anti-TNF-α or anti-IL-1β-neutralizing antibody or its isotype control, were used to stimulate osteoclast precursor RAW 264.7 cells. IL-6 **(A)**, TNF-α **(B)**, and IL-1β **(C)** secretion was determined in culture supernatants by ELISA. NT, non-treated. The graphics are showing values obtained from three independent experiments. Data are given as mean ± SD. ***P < 0.001 *versus* NT. ^##^ *P* < 0.01; ^###^ *P* < 0.001.

To better understand the osteoimmunological features involved in *K. kingae*-induced osteoclastogenesis, experiments were conducted to evaluate the ability of RAW 264.7-derived osteoclast to secrete cytokines in response to culture supernatants from *K. kingae*-infected THP-1 monocytes in the presence or not of neutralizing antibodies. Our results indicated that the addition of culture supernatants from *K. kingae*-infected human monocytes to uninfected murine osteoclast precursors induced a significant secretion of TNF-α, IL-1β, and IL-6 compared with unstimulated cells or cells stimulated with culture supernatants from uninfected monocytes. In addition, blocking of TNF-α and IL-1β significantly reduced the ability of culture supernatants from *K. kingae*-infected human monocytes to induce proinflammatory cytokine secretion, whereas the isotype control had no effect (**Figure 3**). Considering the species specificity of the antibodies used in the ELISA kit for cytokine measurement, the number of secreted factors present in culture supernatants from stimulated murine osteoclast precursors did not cross-react with human cytokines (not shown). These results indicate that the culture supernatants from *K. kingae*-infected monocytes activate proinflammatory cytokine production on osteoclast precursors.

### *K. kingae* Induces Functional Osteoclast Cells

**Figure 4:**
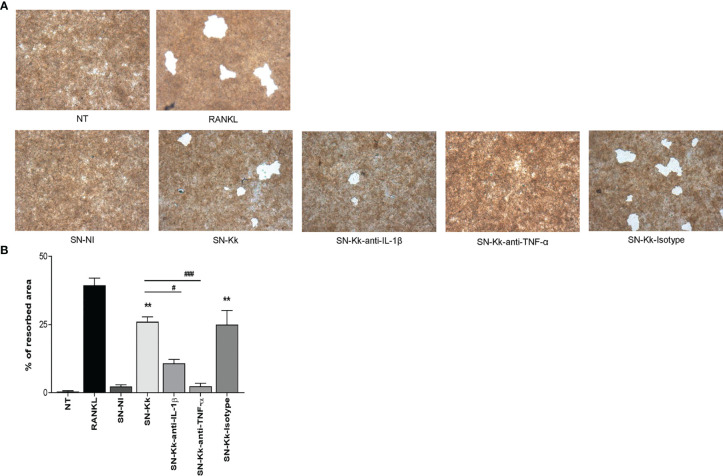
*Kingella kingae* induces functional osteoclasts cells. The functional activity of *K. kingae-*induced osteoclasts was determined by their ability to resorb hydroxyapatite. RAW 264.7 cells were cultured on hydroxyapatite disks under the same conditions as described above. After 7 days, the cells were removed, hydroxyapatite resorption was determined by light microscopy **(A)**, and the resorbed area was quantified (% of resorbed area) **(B)**. The graphics are showing values obtained from three independent experiments. Data are given as mean ± SD. *P < 0.05; **P < 0.01 *versus* cells treated with SN-NI or NT. ^##^ *P* < 0.01; ^###^ *P* < 0.001.

To elucidate whether *K. kingae* infection can generate a microenvironment favoring osteoclastogenesis and bone loss, we have assessed the functional activity of *K. kingae*-induced osteoclast-like cells by their ability to resorb hydroxyapatite. The *K. kingae*-infected human monocytes were able to induce significant hydroxyapatite resorption. In contrast, culture supernatants from uninfected cells did not (**Figure 4**). Additionally, the resorption of hydroxyapatite was significantly inhibited either by anti-TNF-α antibody or by anti-IL-1β antibody but not by the isotype control. These results together indicate that *K. kingae*-infected monocytes released cytokines, such as TNF-α and IL-1β, that promote functional osteoclast formation.

### Pretreatment With *K. kingae*-Derived OMV Decreases the Cytokine Response of Monocytes to *Kingella* Infection

**Figure 5:**
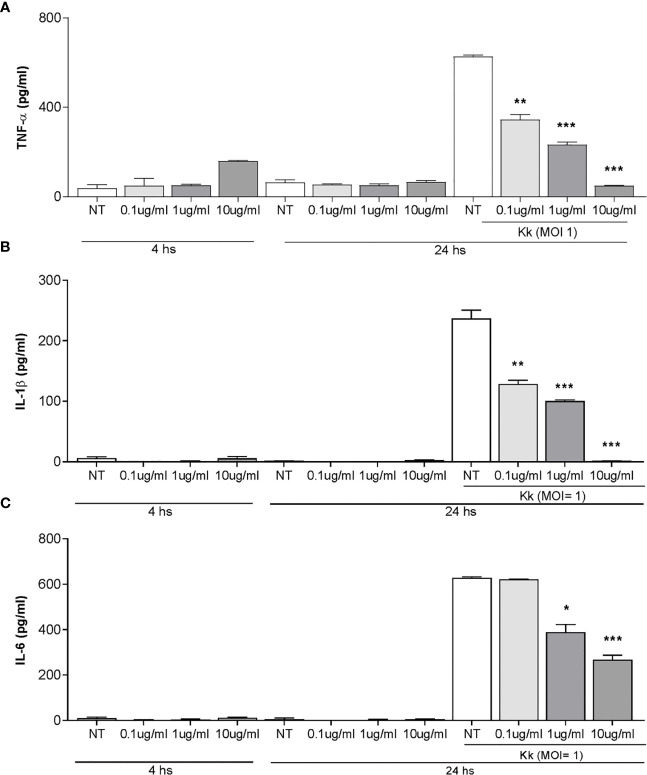
Pretreatment with *Kingella kingae* outer membrane vesicles (OMV) decreases the cytokine response of THP-1 cells to *K. kingae* infection. THP-1 cells were incubated with OMV at a concentration of 0.1, 1, and 10 µg/ml for 4 h as control cells were non-treated. Then, these cells were infected or not with *K. kingae* (multiplicity of infection = 1). Culture supernatants were collected at 4 h post-stimulation with OMV and at 24 h post-infection. TNF-α **(A)**, IL-1β **(B)**, and IL-6 **(C)** were measured by commercial ELISAs in the mentioned culture supernatants. The graphics are showing values obtained from three independent experiments. Data are given as the mean ± SD. *P < 0.05; **P < 0.01; ***P < 0.001 *versus* NT.

OMV from *K. kingae* have been previously implicated in the ability to induce cytokine production from osteoblast and synovial fibroblasts (4). Therefore, experiments were conducted to assess whether stimulation with *K. kingae-*released OMV can modulate the cytokine responses of human monocytes. To this end, cytokine production was measured in culture supernatants from THP-1 cells exposed to 0.1, 1, and 10 μg/ml of OMV. Our results indicated that THP-1 cells exposed to OMV during 4 and 24 h did not induce IL-1β, TNF-α, and IL-6 secretion (**Figure 5**).

OMV have a range of immunomodulatory outcomes to stimulate or suppress immune cell responses through their direct effects on host cells (38). Therefore, experiments were conducted to assess whether pre-exposure with *K. kingae*-derived OMV could modulate cytokine release from human monocytes in response to *K. kingae* infection. As shown in **Figure 5**, the level of TNF-α, IL-1β, and IL-6 released from THP-1 cells pre-treated with OMV was significantly lower than from untreated cells. Such inhibitory effects of OMV appear to be dose dependent (**Figure 5C**). In conjunction, these results indicated that OMV from *K. kingae* could inhibit proinflammatory response monocytes to *K. kingae* infection.

### Pretreatment With OMV Decreases Osteoclastogenesis Induced by Supernatants From Monocytes Infected With *K. kingae*

**Figure 6:**
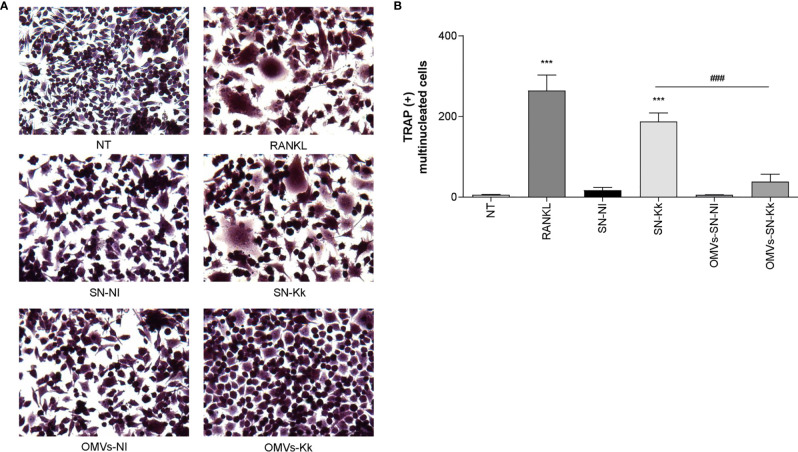
Pretreatment with outer membrane vesicles (OMV) decreases the osteoclastogenesis induced by supernatants from monocytes infected with *Kingella kingae*. RAW 264.7 cells were stimulated with culture supernatants from *K. kingae*-infected THP-1 monocytes (multiplicity of infection = 1) pretreated with OMV (10 µg/ml; OMV-SN-Kk) or with culture supernatants from non-infected THP-1 monocytes pretreated with OMV (OMV-SN-NI) or with supernatants non-pretreated with OMV (SN-Kk and SN-NI, respectively) added at a 1:2 proportion in conjunction with macrophage colony-stimulating factor. After 7 days, osteoclastogenesis was determined by the generation of TRAP-positive multinucleated cells (more than three nuclei). Representative digital images were taken by light microscopy **(A)**, and TRAP-positive multinucleated cells were identified and counted **(B)**. RANKL was used as a positive control. NT, non-treated. The graphics are showing values obtained from three independent experiments. Data are given as mean ± SD. ***P < 0.001 *versus* cells treated with SN-NI or NT. ^###^ *P* < 0.001.

Our results indicate that OMV are crucial for inflammatory responses induced by *K. kingae*. To assess the role of OMV in subsequent osteoclastogenesis induction in response to *K. kingae* infection, osteoclast differentiation was performed in the presence of M-CSF and culture supernatants from *K. kingae*-infected monocytes. Osteoclastogenesis was induced by culture supernatants obtained from *K. kingae*-infected human monocytes, but it was abrogated when cultured human monocytes were exposed to OMV before *K. kingae* infection (**Figure 6**). Additionally, culture supernatants from uninfected monocytes treated with OMV were unable to induce osteoclastogenesis (**Figure 6**). Collectively, these results indicate that cytokine response from human monocytes to *K. kingae* exposure may be conditioned by the OMV coexistence, even modulating its effects on osteoclastogenesis.

### An Increase of OMV/Bacteria Ratio Is Related to the Reduction of Proinflammatory Microenvironment and Inhibition of Osteoclast Differentiation

Live bacteria could contribute to OMV release during *in vitro* infection experiments. To measure the OMV capability to downregulate the osteoclastogenesis, human monocytes were incubated with different doses of OMV prior to HKKk exposure, and then the supernatants were collected for osteoclastogenesis experiments.

**Figure 7:**
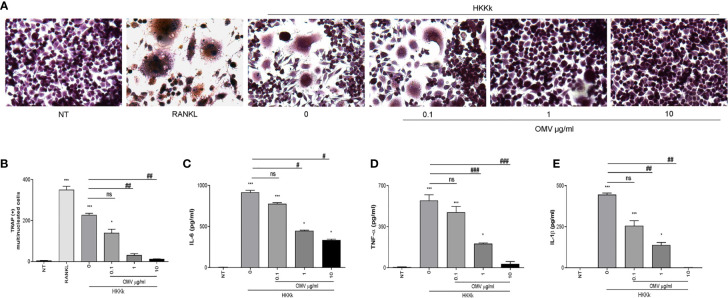
Outer membrane vesicles (OMV) pretreatment decrees osteoclastogenesis induced by HKKk in a dose-dependent manner. RAW 264.7 cells were stimulated with culture supernatants from HKKk-stimulated THP-1 monocytes (1 × 10^6^ bacteria/ml) pretreated with OMV (0, 0.1, 1, and 10 µg/ml) added at a 1:2 proportion in conjunction with macrophage colony-stimulating factor. After 7 days, osteoclastogenesis was determined by the generation of multinucleated TRAP-positive cells (more than three nuclei). Representative digital images were taken by light microscopy **(A)**, and TRAP-positive multinucleated cells were identified and counted **(B)**. RANKL was used as a positive control. NT, non-treated. IL-6 **(C)**, TNF-α **(D)**, and IL-1β **(E)** were measured by commercial ELISAs in culture supernatants from THP-1 cells collected at 24 h post-stimulation. Graphics are showing values obtained from three independent experiments. Data are given as mean ± SD. *P < 0.05; ***P < 0.001 *versus* NT. ^#^ *P* < 0.05; ^##^ *P* < 0.01; ^###^ *P* < 0.001. ns, non-significant.

As shown in **Figure 7**, the osteoclastogenesis was lower as the OMV dose was higher. In concordance, when monocytes were incubated with OMV before HKKk treatment, the secretion of IL-1β, TNF-α, and IL-6 was significantly lower than cells not pretreated with OMV. These results together indicate that *K. kingae* infection could have an opposite action on osteoclast differentiation which is dependent on the bacteria–OMV ratio.

### *K. kingae* Infection Induces Osteoclast Differentiation

**Figure 8:**
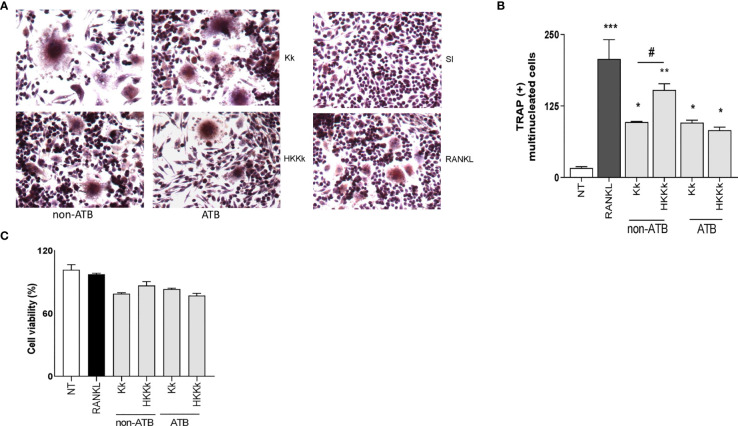
*Kingella kingae* induces osteoclast differentiation. RAW 264.7 cells were infected with *K. kingae* (multiplicity of infection = 1) and HKKk (1 × 10^5^ bacteria/ml) added in conjunction with macrophage colony-stimulating factor without antibiotics (non-ATB). In some wells, the cells were washed, and new culture medium containing antibiotics was added after 2 h (ATB). After 7 days, osteoclastogenesis was determined by the generation of TRAP-positive multinucleated cells (more than three nuclei). Representative digital images were taken by light microscopy **(A)**, and TRAP-positive multinucleated cells were identified and counted **(B)**. Cell viability was measured by MTT colorimetric assay; results were expressed as percent of control (NT cells) **(C)**. RANKL was used as a positive control. NT, non-treated. Graphics are showing values obtained from three independent experiments. Data are given as mean ± SD. *P < 0.05; ***P < 0.001 *versus* NT. ^#^ *P* < 0.05.

The osteoarticular damage associated to *K. kingae* infection may also involve direct pathogenic mechanisms. To elucidate this contribution to osteoclastogenesis, the following experiments were carried out. First, cultured cells were infected with live *K. kingae* or stimulated with HKKk and maintained in the presence of M-CSF, without antibiotics. Second, the cultured cells were challenged as previously but during 2 h and then were washed and maintained in the presence of antibiotics. In both conditions, *K. kingae* infection was able to induce osteoclast differentiation (**Figure 8**). However, when osteoclast differentiation was performed in the absence of antibiotics, the number of osteoclasts from cells treated with HKKk was significantly higher than those produced by live bacteria. The RAW 264.7 cell viability of *K. kingae*-infected ones or from those treated with HKKk was well preserved (80–90%). These results show that *K. kingae* can directly promote osteoclastogenesis beyond its viability. Paradoxically, the osteoclastogenic capability of live bacteria is lower than that of the heat-killed ones, thus insinuating that viable bacteria-released factor/s may downregulate it.

### An Increase of OMV/Bacteria Ratio Reduces Osteoclastogenesis

**Figure 9:**
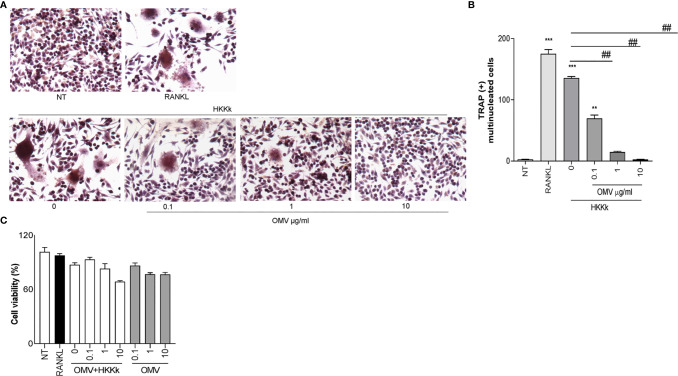
Outer membrane vesicles (OMV) inhibits osteoclast differentiation induced by HKKk. RAW 264.7 cells were stimulated HKKk (1 × 10^5^ bacteria/ml) pretreated with OMV (0, 0.1, 1, and 10 µg/ml) in conjunction with macrophage colony-stimulating factor. After 7 days, osteoclastogenesis was determined by the generation of multinucleated TRAP-positive cells (more than three nuclei). Representative digital images were taken by light microscopy **(A)**, and TRAP-positive multinucleated cells were identified and counted **(B)**. Cell viability was measured by MTT colorimetric assay; results were expressed as percent of control (NT cells) **(C)**. RANKL was used as a positive control. NT, non-treated. The graphics are showing values obtained from three independent experiments. Data are given as mean ± SD. **P < 0.01; ***P < 0.001 *versus* NT. ^##^ *P* < 0.01.

Since HKKk induces higher levels of osteoclastogenesis than live bacteria, we have examined the role of OMV in such osteoclast differentiation. To this aim, RAW264.7 cells were pretreated with different doses of OMV and then treated with HKKk. The OMV diminished osteoclastogenesis in a concentration-dependent manner (**Figure 9**), but they were unable to induce osteoclastogenesis (not shown). These results together insinuate that *K. kingae*-mediated osteoclastogenesis may be counteracted directly by their OMV in a dose-dependent manner. The viability of RAW 264.7 cells exposed to HKKk and treated with OMV was preserved (80–90%).

## Discussion

*K. kingae* mainly produces osteoarticular infections, including septic osteomyelitis and arthritis which cause bone and joint damage (2). While the clinical and imaging features of the osteoarticular disease produced by *K. kingae* have been widely described (8), the pathogenic mechanisms of bone loss have not been elucidated at the cellular and molecular levels. Different cell types are involved in bone resorption, including macrophages, which are pivotal ones not only by the secretion of proinflammatory cytokines but also by differentiating into osteoclasts (33).

Here we report a dual contribution of *Kingella kingae* to promote osteoclastogenesis using direct and indirect mechanisms. About the former, we have observed that *K. kingae* can directly propitiate macrophage differentiation to osteoclasts after an infection or even after exposure, thus inferring that bacteria viability is not an indispensable condition.

Under pathological conditions, the increase of proinflammatory cytokines and RANKL—a key molecule involved in osteoclast differentiation—could contribute to bone injury, encouraging osteoclastogenesis (20–29, 33, 39, 40). Therefore, such differentiation toward osteoclasts was also promoted indirectly by the secreted inflammatory mediators released by bacteria-challenged macrophages. The osteoclasts generated from precursor cells were phenotypically characterized by the generation of TRAP-positive multinucleated cells as well as functionally by the capability of these cells to induce hydroxyapatite resorption.

Interestingly, the two observed osteoclastogenic *K. kingae*-mediated pathways are counteracted by the bacteria-released OMV in a dose-dependent manner. Hence, it is tempting to speculate that *in vivo*, when environmental conditions propitiate OMV production, the tissue damage will be probably limited, while those that diminish OMV will likely increase the tissue damage by enhancing the osteoclastogenic activity. OMV also inhibit proinflammatory cytokine secretion by infected macrophages. It is in contrast with the previous report, in which *K. kingae*-derived OMV can promote a proinflammatory microenvironment by releasing IL-6 and granulocyte–M-CSF secretion from fibroblast, synoviocytes, and osteoblasts (4). The plausible dual OMV role in opposite outcome has been extensively reported for other Gram-negative bacteria (41–46). However, differences in *K. kingae* strain and/or in the preparation of OMV could also be implicated (4).

Both direct and indirect mechanisms of *K. kingae*-mediated osteoclastogenesis do not require bacterial viability, suggesting that osteoarticular damage could involve several virulence factors, such as structural bacterial components or even the RTX (RtxA) toxin. RtxA appears to be secreted in the extracellular environment in a soluble form and probably as a component of the OMV (4). RtxA toxin could participate in bacterial pathogenesis during extravasation of the airway epithelial respiratory barrier and could also contribute to synovium damage and the production of inflammatory mediators (13). The toxic effect of RtxA on THP-1 cells was demonstrated in previous studies (5); however, a non-significant toxic effect was detected in this study. This may be explained by the several differences that display monocyte and macrophage differentiated from THP-1 cells and the time of evaluation of cell death after *K. kingae* infection.

Our data revealed that *K. kingae* is capable of inducing bone destruction mediated by osteoclast independently of the osteoblast contribution. Nevertheless, other models deserve to be developed to determine the role of each bone cell type in bone damage during the infection. The interaction of bone cells during *in vivo* bone loss is not reached in our *in vitro* model. This is considered a limitation of our data. The development of a juvenile rat model was a significant contribution for examining *K. kingae* virulence (15); however, no *in vivo* septic arthritis model is able to determine the contribution of each cell type, the bacteria, and its OMV in the promotion of bone destruction.

In summary, here the results presented a novel pathogenic mechanism of osteoarticular disease triggered by the interplay between the direct and indirect effects of *K. kingae* on osteoclastogenesis and bacteria-released OMV in the opposite direction.

Finally, the present study constitutes the first analysis on osteoclastogenesis alterations during *K. kingae* infection. The role of OMV as a modulator of direct infection and the inflammatory response provide an initial background for more detailed studies as a possible target of therapeutic interventions.

## Data Availability Statement

The raw data supporting the conclusions of this article will be made available by the authors, without undue reservation.

## Author Contributions

AP, FS, CL, and RF performed the experiments. All authors analyzed the data. MD wrote the article. MD and JQ designed the experiments, revised the article, and obtained research funding. All authors contributed to the article and approved the submitted version.

## Funding

This work was supported by grants from Agencia Nacional of Promoción Científica y Tecnológica (ANPCYT, Argentina): PICT 2014-1111, PICT 2015-0316, and PICT 2017-2859 to MD and PICT-2015-1921 to JQ. The funding agency had no role in study design, data collection and analysis, decision to publish, or preparation of the manuscript.

## Conflict of Interest

The authors declare that the research was conducted in the absence of any commercial or financial relationships that could be construed as a potential conflict of interest.

## Publisher’s Note

All claims expressed in this article are solely those of the authors and do not necessarily represent those of their affiliated organizations, or those of the publisher, the editors and the reviewers. Any product that may be evaluated in this article, or claim that may be made by its manufacturer, is not guaranteed or endorsed by the publisher.
